# Supplementary material for: Differential Affinity Chromatography Coupled to Mass Spectrometry: A Suitable Tool to Identify Common Binding Proteins of a Broad-Range Antimicrobial Peptide Derived from Leucinostatin
Source: Biomedicines. 2022 Oct 23;10(11):2675. doi: 10.3390/biomedicines10112675 (PMC9687860; doi:10.3390/biomedicines10112675)
Supplement: Supplementary file 1 [file biomedicines-10-02675-s001.zip › Supplemental_figures.pdf]

Supplemental Figures

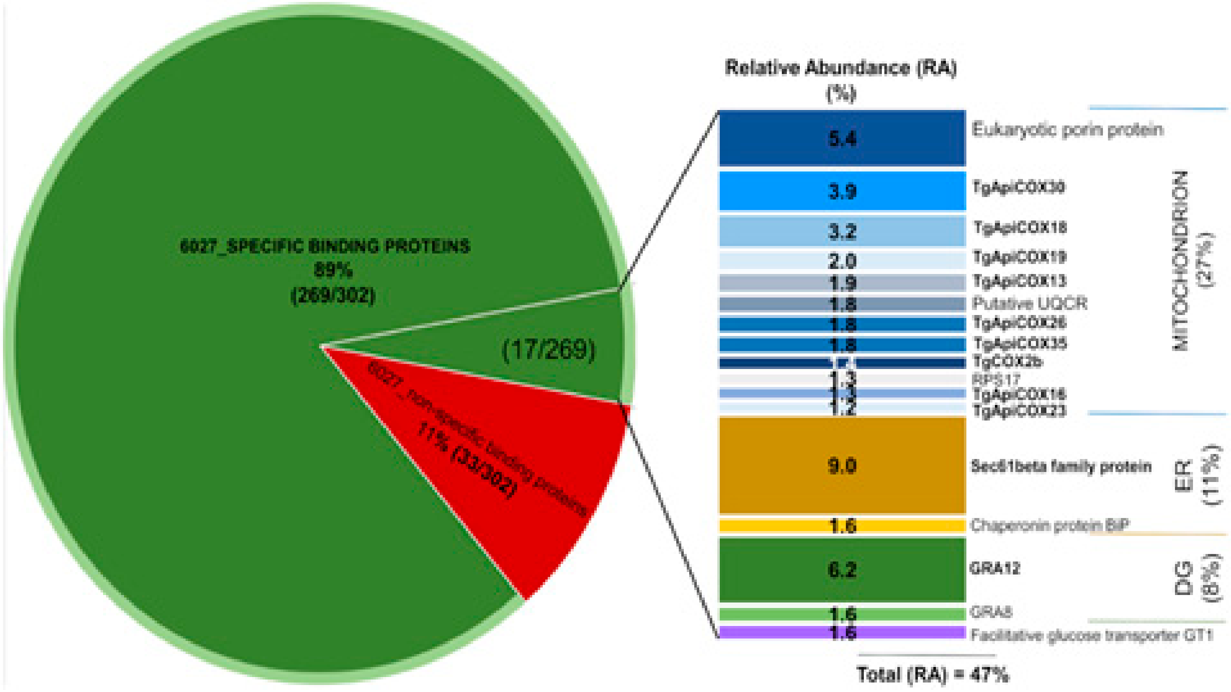

Figure S1. Schematic representation of the most abundant *T. gondii* peptide 6027-binding proteins. Three main clusters are identified that account for 47% relative abundance, assigned to mitochondrion, ER and GRA proteins.

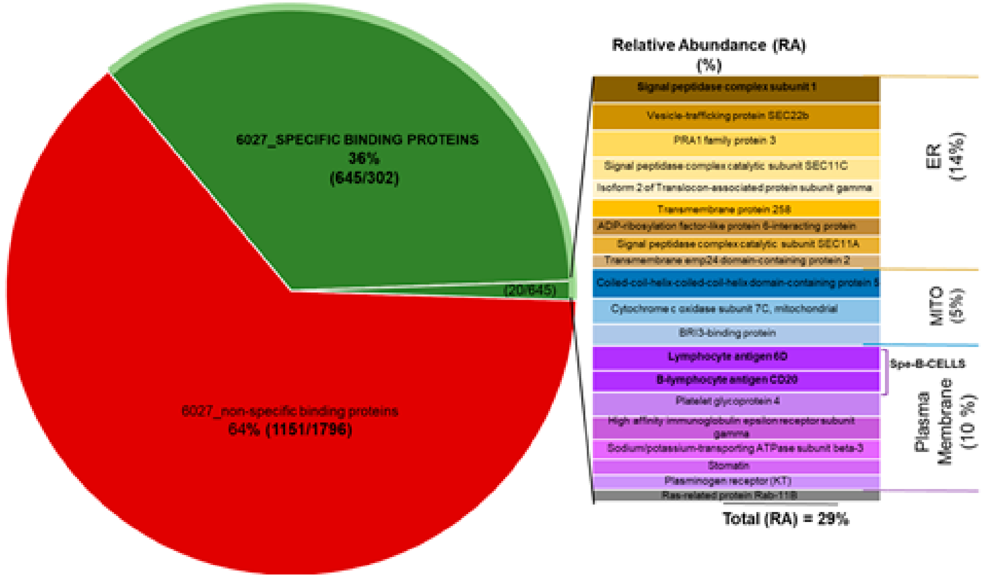

Figure S2. Schematic representation of the most abundant peptide 6027-binding proteins in murine spleen cell extracts. Three main clusters are identified that account for 29% relative abundance, and are assigned to mitochondrion, ER and GRA proteins.
